# Possession and Usage of Insecticidal Bed Nets among the People of Uganda: Is BRAC Uganda Health Programme Pursuing a Pro-Poor Path?

**DOI:** 10.1371/journal.pone.0012660

**Published:** 2010-09-10

**Authors:** Syed Masud Ahmed, Abebual Zerihun

**Affiliations:** 1 Research and Evaluation Division, BRAC, Dhaka, Bangladesh; 2 Evaluation and Research Unit, East Africa Programmes, BRAC, Kampala, Uganda; Kenya Medical Research Institute, Kenya

## Abstract

**Background:**

The use of insecticidal bed nets is found to be an effective public health tool for control of malaria, especially for under-five children and pregnant women. BRAC, an indigenous Bangladeshi non-governmental development organization, started working in the East African state of Uganda in June 2006. As part of its efforts to improve the health and well-being of its participants, BRAC Uganda has been distributing long lasting insecticide-treated bed nets (LLIN) at a subsidized price through health volunteers since February 2008. This study was conducted in March-April 2009 to examine how equitable the programme had been in consistence with BRAC Uganda's pro-poor policy.

**Methodology/Principal Findings:**

Information on possession of LLINs and relevant knowledge on its proper use and maintenance was collected from households either with an under-five child and/or a pregnant woman. The sample included three villages from each of the 10 branch offices where BRAC Uganda's community-based health programme was operating. Data were collected by trained enumerators through face-to-face interviews using a hand-held personal digital assistant (PDA). Findings reveal that the study population had superficial knowledge on malaria and its transmission, including the use and maintenance of LLINs. The households' rate of possession of bed nets (41–59%), and the proportion of under-five children (17–19%) and pregnant women (25–27%) who reported sleeping under an LLIN were not encouraging. Inequity was observed in the number of LLINs possessed by the households, in the knowledge on its use and maintenance, and between the two programme areas.

**Conclusions/Significance:**

The BRAC Uganda's LLINs distribution at a subsidized price appeared to be inadequate and inequitable, and BRAC's knowledge dissemination is insufficient for initiating preventive actions such as proper use of LLINs to interrupt malaria transmission. Findings contribute to the on-going debate on LLINs distribution in Africa and make a strong case for its free distribution.

## Introduction

Malaria is a public health problem in some 90 countries worldwide affecting at least 300 million people [1]. It is estimated to be directly responsible for about one million deaths annually or 3,000 deaths a day worldwide, 90% in Africa—mostly at homes [2]. Recent global initiatives to control malaria include a combination of preventive and curative measures such as vector control, use of bed nets, mosquito repellants, chemoprophylaxis, and effective case management [3]–[5]. Among the preventive measures, the use of insecticidal bed nets such as LLIN (Long Lasting Insecticide-treated Nets) is found to be an effective public health tool for control of malaria, especially among under-five children and pregnant women — the two most vulnerable groups [6]–[10]. This has been compared with generation of ‘herd immunity’ as in the case of vaccines. For this the coverage has to be ‘sufficiently high’ (say, beyond 80%). To achieve this high coverage, mass distribution of insecticidal nets is recommended [11]. Also, to make the coverage equitable, free distribution is advocated [12], though argument favouring a ‘for-profit’ approach also exists [13]. However, when insecticidal bed nets are distributed free of cost instead of cost recovery or (heavily) subsidized cost approach, evidence from 40 malaria-endemic African countries shows that the coverage becomes more equitable [14], and also, rapidly scalable [15].

Malaria is the major cause of illness and death in children in Uganda and is responsible for 25% to 30% of under-five deaths, resulting in 70,000 to 100,000 deaths annually countrywide [16]. Over 90% of the population live in high endemic areas with perennial transmission, while 10% live in low transmission areas which are prone to malaria epidemics [17]. Children under five years and pregnant women are most vulnerable to malaria, yet only 28% of children under-five sleep under a bed net.

BRAC, an indigenous Bangladeshi non-governmental development organization (NGO) working for poverty alleviation and empowerment of the poor, especially women ((http://www.brac.net), launched its much lauded model of microfinance plus programme in the East African state of Uganda in June 2006 [18]. As part of its effort to improve the health and well-being of the population, BRAC Uganda has been distributing LLINs through volunteer Community Health Promoters (CHP) under its Essential Health Care (EHC) programme since February 2008 to protect pregnant women and under-five children especially against malaria. BRAC Uganda initially sold the nets at market price. However, to boost sales among the poor, it subsequently started selling the nets at a subsidized price. This study was done to examine whether this new approach reached the poor and vulnerable groups, in consistence with BRAC Uganda's pro-poor strategy.

## Methods

### Ethics statement

The study passed through the institutional review process at BRAC Research and Evaluation Division (Internal Review and Publication Committee) for ethical approval. No invasive procedure was done. Informed verbal consents were obtained from the respondents who were skeptical about signing any document. The written consent form was read out and explained to the respondents and when the investigator was satisfied that the respondent understood it including its implications, and had agreed to participate, only then was she included in the survey. Anonymity of the respondents was maintained at all stages of data analysis.

### The intervention

BRAC Uganda started its essential health care (EHC) programme in 10 branches in Kampala area (Kampala and Mukono) and eastern districts (Jinja, Iganga, Bugiri, and Busia districts). These branches were usually located in the sub-counties and parishes, and each branch operates in 20–30 villages located within 4 kilometers radius of the branch office. The LLINs were distributed through the community health promoters (CHP) resident in each village under the direct supervision of the community health assistants (CHA) at the branch office.

The CHPs are health volunteers who received 15 days residential training by BRAC Uganda before deployment in the villages, and their initial training is backed up by monthly refreshers. Besides being a reliable source of low-cost health products such as LLINs and anti-malarials, the CHPs also disseminate health education messages including messages on the mode of transmission of malaria, role of insecticidal bed nets to interrupt the cycle of transmission, and proper use and maintenance of the bed nets. For the latter, the CHPs use a number of approaches such as inter-personal communication during household visits, giving talks at different forums (e.g., micro-finance borrowing groups, women's ‘good health’ forums, congregation at schools, etc.), setting up ‘health stalls’ at local markets during market days, and maintaining daily ‘open hours’ at their residence or some designated place in the village. Each day a CHP conducts door-to-door visits to10 households, and to 180–200 households in a month while health forums take place once a week.

The bed nets were supplied to the CHPs in the form of a revolving fund worth UGX 100,000 (∼US$50) which included other health commodities as well, given to the CHPs initially in the form of a loan. To start with, each CHP received one bed net at a time, but she could procure more bed nets as often as necessary once she sold and refunded the loan from the sale proceeds. The CHPs purchased the LLINs at a price of UGX 10,500 (∼US$5) and sold at UGX 12,000 (∼US$6). During January 2008 to December 2009, a total of 2,131 LLINs were sold by the CHPs.

### Design

This cross-sectional quantitative survey compared the two areas where BRAC EHC programme was in operation. As no baseline data were available, a post-test only design was adopted and comparison was made between the two areas of programme implementation (e.g., peri-urban and rural areas) to explore the existence of differences, if any. The latter was also of interest to the programme managers from operational aspect. The coverage and use data for bed nets were compared with WHO recommended standards which stipulate ‘to ensure that at least 80% of those at risk of, or suffering from, malaria should benefit from major preventive and curative interventions by 2010’ [19].

### Sampling

Due to constraints in time and resources, a purposive sampling technique was used to select study households. From each of the 10 branches, three villages were randomly chosen where insecticidal bed nets had been distributed by the programme (total villages = 30). Data were collected from each households of these villages that had either one under-five child and/or a pregnant woman, each household being included only once. It may be mentioned here that no other agency had distributed insecticidal bed nets in these villages earlier.

### The survey

The survey was undertaken during March–May 2009, which coincided with the rainy season and also the first spell of malaria season (March–June) in Uganda. Data on socio-demographic characteristics, knowledge on malaria, and possession and usage of LLINs were collected through face-to-face interviews with either the household head or spouse of the eligible households. Trained interviewers used hand held computers (Personal Digital Assistant or PDA) to collect and store data. The survey team was selected from a pool of graduate level enumerators who have worked with BRAC Uganda in various large scale surveys and are experienced in using a PDA.

The feasibility of PDA-based survey was piloted by BRAC Uganda Evaluation and Research Unit. Pre-testing was done for technological feasibility, logistic requirements, consistency and appropriateness of language of the questionnaire. For every five enumerators, one technical person specializing in PDA was hired to work as supervisor cum on-the-spot trouble-shooter. Moreover, the supervisors were responsible for synchronizing the data on a daily basis from the PDAs to the computers set up at branch offices for survey purpose. The overall technical supervision was done by a Senior Technology Specialist (STS), who was BRAC Uganda's full-time employee based at the Evaluation and Research Unit.

In each village, the survey team selected and interviewed all eligible households. First, the supervisor identified land marks which were used as the starting point. Interviewers then walked along strictly regulated routes following the ‘left hand rule’, turning left at every junction of the road, track or pathway and interviewing every household that has at least one child under five years of age and/or a pregnant woman. This was done to ensure that there was no bias in household selection. The day-to-day field activities of the teams were closely monitored by the field researchers. To ensure quality of data collected, independent teams spot-checked households randomly within three days of the main survey. In cases where inconsistencies were noted, relevant interviewers were accompanied by a field supervisor for a re-interview until quality standards were met.

Data were cleaned and analysed using SPSS ver 12. Comparison was made between peri-urban (Kampala) and rural (eastern districts) areas, and wealth quintiles (poorest vs. least poor) to address equity issues.

### The variables

When individuals living together took a meal from a common cooking facility, the entity is defined as a household HH. The head is defined as the person who was perceived by HH members to be the primary decision-maker in the family and who may or may not had been the main income-earner. Education was measured by completed years of formal schooling. Engagement in a particular income-earning activity for the major part of the day was categorized as the ‘main occupation.’

Socioeconomic stratification (SES) of the households was done based on the possession of different types of asset. Respondents were asked about 30 types of assets (homestead, goats/sheep, TV, cell phone, clock/watch, bicycle, toilet, etc). Thus, an asset index was constructed using factor analysis. Eleven assets were carefully examined and included in the asset index based on their strength of correlation (0.3 and above) and an optimal percentage requirement of total variance explained (30%). Thus, the final list included variables such as homestead ownership, type of roof, floor and wall material, source of drinking water, ownership of phone, bicycle, cattle/cows, sheep/goats, chickens and ducks. Ownership of chickens, ducks, sheep/goats, homestead, and bicycle were found to be the strongest indicators. This component explained 31% of the variations in the selected 11 indicators. To check the robustness of these indicators, we ran KMO and Bartlett's test of sphericity which was found to be 0.89. The asset scores were further classified into five quintiles, starting from the lowest (1^st^ quintile, poorest) to highest (5^th^ quintile, least poor).

## Results

### Household characteristics

The average household size of respondents was four and six respectively in the Kampala and eastern districts areas (Table 1), compared to five found in the 2006 DHS [17]. Around one-fifth of the households were headed by females. There were more pregnant women in the eastern districts areas (34% vs 22% in Kampala areas) and more under-five children in the Kampala areas (78% vs 66% in eastern district areas). Self-rated chronic deficit households were present in greater proportion in the eastern district areas (10%) than in the Kampala areas (6%). The majority of the household heads (around 85%) had formal education and the most common occupation was non-agriculture self employment and small trade (50–60%) (Table 1).

**Table 1:** Socio-demographic characteristics of the respondents by programme areas and sex (%).

	Peri-urban (Kampala and Mukono) areas	Rural (Eastern Districts) areas*
Household Characteristics		
Household size	4.3	5.6
Female-headed households	23.5	18.5
HH having at least one pregnant woman	22.3	33.9
HH having at least one under- five child	77.7	66.1
Household head's highest level of schooling		
None	16.7	12.8
Primary Level	37.8	29.0
> Primary Level	45.5	58.2
Household head's main occupation		
Farming	2.0	17.8
Wage labor	22.8	14.9
Non-agricultural self-employment	59.1	51.0
Government/private Service	15.9	16.3
Household asset quintiles		
Poorest (1^st^ Quintile)	1.7	57.9
3^rd^ Quintile	37.8	8.7
Least Poor (5^th^ Quintile)	40.9	0.9
Self-rated poverty status of HH		
Chronic deficit	6.2	10.3
Occasional deficit	21.1	30.4
Break-even/No deficit	66.1	56.9
N	3,248	6,986

*Eastern Districts areas include Jinja, Iganga, Bugiri and Busia districts.

### Knowledge on malaria, its prevention and treatment

Respondents were asked about the cause, modes of transmission, prevention and treatment of malaria. The knowledge that malaria is caused by ‘mosquito bite’ was nearly universal, but the majority (60–65%) did not know which type of mosquito causes it, and only around a third correctly knew that it is transmitted ‘by bite of mosquito which has bitten a malarial patient’ (Table 2). Respondents belonging to the poorest quintile were disadvantaged in this aspect. Interestingly, use of insecticide impregnated bed nets (e.g., LLINs) as a preventive measure was mentioned by less than 2% of the respondents while regular bed nets was mentioned by a much greater proportion (46–79%). For treatment of malaria, the government hospital was mentioned more frequently by the ‘poorest,’ while private clinic was mentioned more frequently by the ‘least-poor,’ with the difference being larger in the Kampala areas (Table 2).

**Table 2:** Reported knowledge on malaria by study areas and wealth quintiles (multiple responses) (%).

	Peri-urban (Kampala and Mukono) areas			Rural (Eastern Districts) areas*		
	Poorest	3^rd^ quintile	Least poor	Poorest	3^rd^ quintile	Least poor
Causes of malaria						
Mosquito bite	93.3	93.2	93.6	95.4	97.4	96.9
Fly/Insect bite	1.1	2.6	2.3	1.0	0.8	1.0
Lack of cleanliness	5.6	4.0	3.5	3.6	1.8	2.1
χ^2^ Significance		p<0.001			ns	
Mode of transmission						
By bite of any mosquito	60.5	60.9	57.9	71.7	65.3	58.0
By bite of mosquito which has bitten a malaria patient	33.7	32.6	35.7	20.7	29.2	34.2
Other	5.8	6.5	6.4	7.6	5.6	7.8
χ^2^ Significance		p<0.05			p<0.001	
Mode of Prevention						
Preventing breeding of mosquito	28.7	36.5	50.3	12.5	25.9	51.9
Using bed net	64.3	59.0	46.1	79.0	69.2	45.4
Using Mosquito repellent/coil	4.9	2.3	1.2	7.2	3.8	1.5
Using insecticide impregnated nets	1.1	1.8	1.8	1.4	1.1	1.2
χ^2^ Significance		p<0.001			p<0.001	
Mode of Treatment						
Government Hospital	51.3	41.2	33.9	75.7	62.5	50.1
Private health Center	27.5	38.3	50.0	13.0	23.3	28.8
Village Doctor	7.5	7.2	10.1	0.5	2.2	11.2
Drug Seller	13.4	12.8	6.0	10.9	12.0	10.0
χ^2^ Significance		ns			p<0.001	
N	671	461	174	1483	1416	2084

*Eastern Districts areas include Jinja, Iganga, Bugiri and Busia districts.

### Insecticidal bed nets (LLIN) possession, usage, and knowledge on norms of use

Findings about insecticidal bed net possession are presented in Table 3. A greater proportion of households from Kampala areas possessed at least one LLIN compared to eastern districts areas (59% vs. 41%, p<0.01), with no variation by wealth quintiles in the Kampala areas and marginal variation in Eastern Districts areas. However, the mean number of LLINs possessed showed variation by SES disfavouring the poorest households, especially in the Kampala areas (Table 3).

**Table 3:** Possession of Long Lasting Insecticide-treated bed Nets (LLINs) by programme areas & wealth quintiles.

	Peri-urban (Kampala and Mukono) areas				Rural (Eastern Districts) areas*				
	Poorest	3rd quintile	Least poor	All	Poorest	3rd quintile	Least poor	All	t-test**
% HHs with at least one LLIN	58.3	60.3	58.6	59.0	42.3	44.0	37.5	40.9	p<0.01
Mean No. of LLIN per household	1.6	2.4	2.6	1.9	1.7	2.1	2.5	2.1	ns
% of HH members who slept under LLINs in the previous night	16.0	23.6	26.4	16.5	21.4	22.7	18.1	13.1	ns
% of under -five children who slept under LLINs in the previous night	14.0	18.0	19.0	18.9	18.3	17.7	14.7	17.0	p<0.10
% of pregnant woman who slept under LLINs in the previous night	2.1	6.9	5.6	24.8	3.9	6.9	5.1	26.9	p<0.01
N	671	461	174	1306	1483	1416	2084	4983	

*Eastern Districts areas include Jinja, Iganga, Bugiri and Busia districts.

**Kampala vs. Eastern districts.

When investigated about the use of LLINs by the household members (13–16%), the under-fives (17–19%) and the pregnant women (25–27%) in the night before the day of survey, the ‘poorest’ households were found to be clearly disadvantaged in the Kampala areas but not in the eastern districts areas (Table 3). However, under-five children received preferential treatment over pregnant women while sleeping under an insecticidal bed net, irrespective of SES. More than half of the households hung the LLINs at the recommended time (‘just before evening sets in’) in Kampala areas (53%), but far less in the eastern districts areas (36%), again without substantial difference by SES (Table 4).

**Table 4:** Hanging insecticidal bed nets by programme areas & wealth quintiles.

	Peri-urban (Kampala and Mukono) areas				Rural (Eastern Districts) areas*			
	Poorest	3^rd^ quintile	Least poor	All	Poorest	3^rd^ quintile	Least poor	All
% HH that hanged LLINs								
just before evening set in	45.3	41.6	42.2	53.1	24.0	23.4	18.3	36.6
before sleep at night	54.7	58.4	57.8	46.9	76.0	76.7	81.7	63.7
χ^2^ Significance	p<0.01				ns			
N	671	461	174	1306	1483	1416	2084	4983

*Eastern Districts areas include Jinja, Iganga, Bugiri and Busia districts.

Knowledge on norms of maintenance of the LLINs was alarmingly poor, only 3.3% in Kampala areas and 1.3% in eastern district areas knew all norms of maintenance of LLINs, the better-offs better than the ‘poorest’ households (Table 5). Critical knowledge such as a place for washing the insecticidal bed nets and maximum number of washes annually was mostly lacking.

**Table 5:** Knowledge on norms of using insecticidal bed nets by wealth quintiles (multiple responses) (%).

	Urban (Kampala and Mukono) areas				Rural (Eastern Districts) areas*			
	Poorest	3rd quintile	Least poor	All	Poorest	3rd quintile	Least poor	All
Knows about how to maintain LLINs								
No wash directly in the pond or canal water	6.1	13.3	11.2	14.5	5.0	2.3	6.3	8.2
Not to keep in the sun after washing	20.4	30.1	38.0	44.4	32.5	26.7	22.0	43.7
Three/four washes in a year	0.0	0.9	1.6	1.5	14.4	0.2	0.2	3.4
χ^2^ Significance	p<0.01				p<0.01			
Knows all norms of LLINs maintenance	0.0	5.3	2.0	3.3	0.0	0.2	1.2	1.3
N	671	461	174	1306	1483	1416	2084	4983

*Eastern Districts areas include Jinja, Iganga, Bugiri and Busia districts.

## Discussion

This study was conducted to investigate whether BRAC Uganda's approach to distribute LLINs at subsidized price succeeded in fulfilling its pro-poor strategy. Findings reveal that the study population had superficial knowledge on different aspects of malaria and its transmission, which may be inadequate to take preventive actions. Inequity was observed both in the number of LLINs possessed by the households as well as knowledge regarding its use and maintenance. The implications of these findings for the programme are discussed.

The households' possession of bed nets (41–59%), and the proportion of under-five children (17–19%) and pregnant women (25–27%) sleeping under an LLIN, were not encouraging. Their knowledge regarding the proper time of hanging and maintenance of bed nets (1–3%) was also alarmingly poor. For reaping the ‘herd immunity’ benefit from distribution of insecticidal bed nets, coverage has to be ‘sufficiently high’ (say, above 80%) and for a family size of five, three bed nets are recommended [11]. The programme was far from achieving these targets. However, the vulnerable groups (under-fives and pregnant women) received preferential treatment in sleeping under the LLINs as also observed elsewhere [20].

In the peri-urban (Kampala) areas, the poorest households were especially disadvantaged regarding the possession of LLINs and its preferential use by the vulnerable groups, compared to its least poor counterparts. Thus, BRAC Uganda's strategy of LLIN distribution at subsidized cost appeared to be inequitable particularly in the peri-urban areas. This is further corroborated by recent evidence from 40 malaria-endemic African countries that even heavily subsidized approach fails to ensure equitable distribution compared to free distribution [14]. To make the process equitable, distribution of nets free-of-cost [21], and utilizing visits to health services for preventive services such as vaccination for children [22] or antenatal care for pregnant women [9] are advocated.

The gap between household possession of LLINs (41–59%) and individual use by vulnerable groups (17–27%) observed in this study draws our attention to an important aspect of human behaviour. It has been found that neither the distribution of insecticidal nets nor the knowledge on malaria transmission and prevention automatically translate into its use [23], [24]. The information disseminated needs to be culture-sensitive and based on existing positive beliefs and behavior if it is to be acceptable by the community. This is important because comprehensive knowledge on different aspects of malaria has been found to influence the use of insecticidal bed nets [25]. Thus, the programme should re-align its IEC (Information, Education and Communication)/BCC (Behaviour Change Communications) campaigns on malaria and bed nets to make it culturally-sensitive and therefore improve compliance by the poorer section of the community. Also, the programme urgently needs to address the regional divide observed between the two areas (peri-urban and rural) with respect to hardware (distribution of LLINs) and software (IEC campaigns) components before it becomes a serious problem of inequity.

To conclude, BRAC Uganda should aim at distributing LLINs free-of-cost to cover the marginalized population to make the programme equitable [26]. Engagement of the community in the process will be helpful as observed in the Solomon Islands [27]. Also, the “Catch-up (mass, free distribution)” campaigns should be backed up by the “Keep-up (long-term, routine access to new nets)” process to sustain coverage over time [28]. Mobilizing resources for free distribution of LLINs or retreatment of regular bed nets with insecticides as a stop-gap measure in a scenario of ‘disparities and inadequacies’ in donor funding across Africa [29] pose a serious challenge to BRAC Uganda. This needs to be addressed prudently if rapid and effective scaling up is desired.
